# First Successful Treatment Reported with Pembrolizumab in a Patient Diagnosed with Choriocarcinoma in Hungary

**DOI:** 10.3390/life16030481

**Published:** 2026-03-16

**Authors:** Kornel Fulop Lakatos, László Kalmár, Erika Lahm, Erzsébet Gyöngyvirág Bíró, Vilmos Fülöp

**Affiliations:** 1 Faculty of Health and Sport Sciences, Széchenyi István University, 1 Egyetem ter, 9026 Győr, Hungary; 2 Department of Obstetrics and Gynecology, Central Hospital of Northern Pest-Military Hospital, 44 Robert Karoly korut, 1134 Budapest, Hungary; 3 Department of Oncology, Central Hospital of Northern Pest- Military Hospital, 44 Robert Karoly korut, 1134 Budapest, Hungary; 4 CT-MR Imaging Laboratory, National Institute of Oncology, Radiology, 7-9 Rath Gyorgy utca, 1122 Budapest, Hungary; 5 Faculty of Health Sciences, University of Miskolc, 1 Egyetem ut, 3515 Miskolc, Hungary

**Keywords:** pembrolizumab, choriocarcinoma, immunotherapy

## Abstract

Pembrolizumab is a programmed cell death protein (PD-1) inhibitor, humanized antibody widely used in cancer immunotherapy. Choriocarcinoma is an aggressive type of gestational trophoblastic neoplasia. Its treatment is based on surgical removal of the tumorous tissue and systemic chemotherapy; however, in some chemoresistant cases, immunotherapy can also be a valid option. Here, we report the first successful programmed death inhibitor-based treatment of a patient diagnosed with stage IV, ultra-high-risk choriocarcinoma in Hungary.

## 1. Introduction

Gestational trophoblastic disease (GTD) is a wide group of conditions affecting different types of trophoblastic cell types. Based on the World Health Organization (WHO) classification, the term gestational trophoblastic neoplasia (GTN) covers the conditions that present malignant and invasive tendencies. Before gynecological ultrasound had become a routine practice, the diagnosis of gestational trophoblastic disease was based on analytically confirmed pregnancy and clinical presentation. Due to early ultrasound diagnosis, the molar pregnancies are often diagnosed not later than the 6th–7th week of pregnancy, providing a relatively good chance for the clinician for optimal treatment and full recovery for the patient [1,2]. The treatment consists of surgical evacuation of the uterus. Molar pregnancy can have invasive presentation that requires adjuvant chemotherapy. However, adjuvant chemotherapy is not always necessary when complete surgical removal of the tumor is followed by normal levels of serum human chorionic gonadotropin (hCG) [3]. Based on the WHO score system, low-risk and high-risk GTN are established. Low-risk disease can be treated with mono-agent chemotherapy, most frequently with methotrexate or actinomycin-D, following evacuation of the uterus. High-risk gestational disease is treated with etoposide, methotrexate, actinomycin-D and cyclophosphamide and vincristine (EMA-CO) regime or cyclophosphamide and vincristine substituted for the cisplatin and etoposide regime (EMA-EP) [4]. However, in chemotherapy-resistant cases, immunotherapy should also be considered. There are no standard criteria for the definition of chemoresistant disease, but the most consensual signs are a plateau or increase in the serum hCG-β and the appearance of new metastasis. According to Feng et al., the diagnosis of chemoresistant GTN should include the following: 1. Rising of serum hCG-β after receiving at least two cycles of multiagent chemotherapy; 2. Presenting plateau of serum hCG-β which consists of at least three weakly detected hCG-β levels that have failed to decrease more than 10% below the preceding hCG-β level; 3. Presenting new metastasis or increase in the tumor size during the treatment with the multiagent chemotherapy [5].

Pembrolizumab is a programmed cell death protein (PD-1) inhibitor, humanized antibody widely used in cancer immunotherapy [6]. In case of GTN our criteria for the therapy included that the candidate should present multiagent chemotherapy-resistant disease and either had recurrent or progressive intermediate trophoblastic tumor (placental site trophoblastic tumor or epithelioid trophoblastic tumor) or high-risk disease. PDL-1 positivity of the tumor is also necessary (50% in the presented case).

## 2. Clinical Case

**Figure 1:**
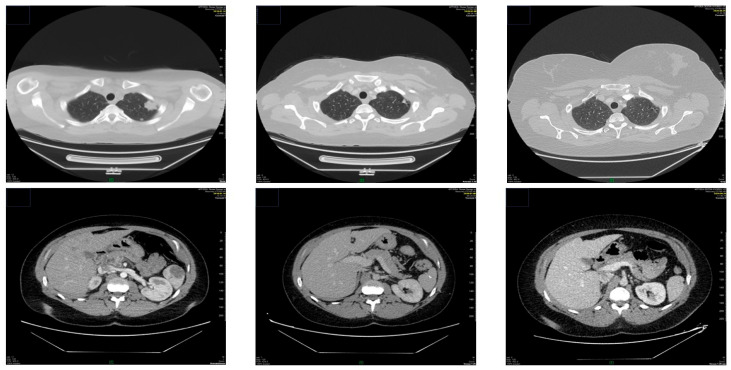
We can see the CT image of the lung metastasis and its complete regression (upper line) and the splenic metastasis and its complete regression. GE Optima^TM^ 660 (GE Health Care, 500 West Monroe Street, Chicago, IL 60661, USA) was used.

This study was approved by the Institutional Review Board protocol of the Central Hospital of Northern Pest. We report a 32-year-old woman (G: 1 P: 1 Ab: 0) who gave birth to a healthy newborn after a high-risk pregnancy presenting gestational hypertension, maternal obesity (BMI > 35 kg/m^2^), and premature rupture of membranes at week 39. The patient was diagnosed with choriocarcinoma after presenting metrorrhagia, hemoptysis and dyspnea 4 months after C-section was performed (September 2018). Histopathology via dilatation and curettage (DLC) confirmed choriocarcinoma and pulmonary, and bone, splenic and pancreatic metastasis were diagnosed by CAT scan and bronchoscopy (see Figure 1). An Etoposide, methotrexate, actinomycin-D, cyclophosphamide, vincristine (EMA-CO) regime (3 cycles 10/2018–11/2018: 200 mg etoposide, 400 mg methotrexate, 1200 mg cyclophosphamide, 2 mg vincristine, 0.5 mg actinomycin-D) and a bleomycin, etoposide, cisplatin/actinomycin-D (BEP/A) regime were used (1 cycle, January 2019: bleomycin: 30 mg, etoposide: 200 mg, cisplatin: 40 mg), followed by transabdominal hysterectomy and bilateral salpingectomy with ovaria spared (1 November 2019). Based on the WHO classification, the patient presented stage IV, ultra-high-risk disease (15 points) as the choriocarcinoma was diagnosed within 4–6 months (1 point) after a term delivery (2 points), the pretreatment serum hCG-β was 274,000 U/L (4 points), 5–8 metastases were detected (2 points), including splenic and pancreatic metastasis (2 points), the largest tumor exceeded 5 cm (2 points), and the number of unsuccessfully used chemotherapy agents was more than 2 (4 points) [7].

**Figure 2:**
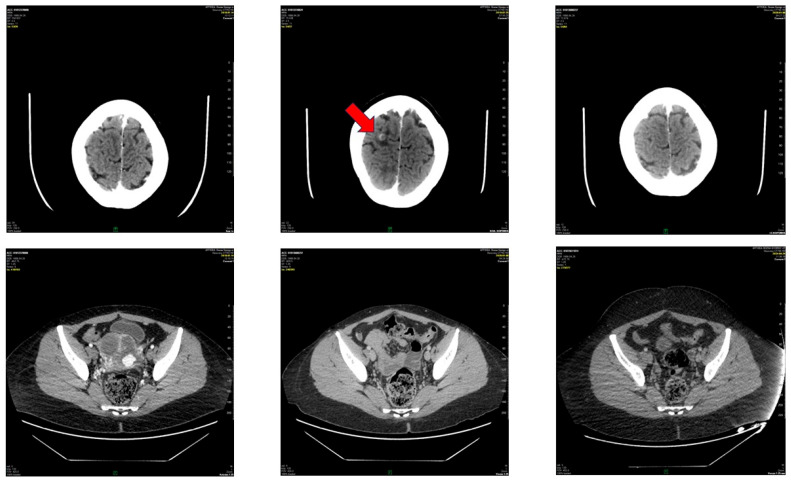
Appearance of brain metastasis can be seen (red arrow), and its complete regression after stereotactic radiotherapy (upper line). CT image of the pelvis with uterus affected by choriocarcinoma before surgery and complete regression of the disease after surgery, multiagent chemotherapy and 62 cycles of pembrolizumab. GE Optima^TM^ 660 (GE Health Care, 500 West Monroe Street, Chicago, IL 60661, USA) was used.

Afterwards, an inter-institutional council was requested from the New England Trophoblastic Disease Centrum, and EP-EMA was started, followed by a paclitaxel, cisplatin, etoposide (TP/TE) regime that led to temporal remission of the disease. Then, 240 mg paclitaxel, 2 mg vincristine and 60 mg cisplatin were given. Due to brain metastasis (see Figure 2), the patient received stereotactic irradiation therapy in the National Institute of Oncology. EMA-CO and TP/TE were re-established (October 2019–January 2020).

Finally, after important re-elevation of serum human chorionic gonadotropin hCG-β and multiplex metastasis, pembrolizumab therapy was started (21 February 2020), supported by the histopathological finding of high programmed death ligand (PDL) membrane positivity 50%, TPS: 90%, CPS = 100. DAKO 22C3 monoclonal antibody was used for immunohistochemistry technique. According to the PMB (pembrolizumab) protocol (97-235862/2020) of the National Health Insurance Fund of Hungary (Hungarian acronym: NEAK), 200 mg of Keytruda^®^ was given in cycles of 21 days. No interruptions were detected. The patient received the 76th cycle of pembrolizumab as of November 2025.

**Figure 3:**
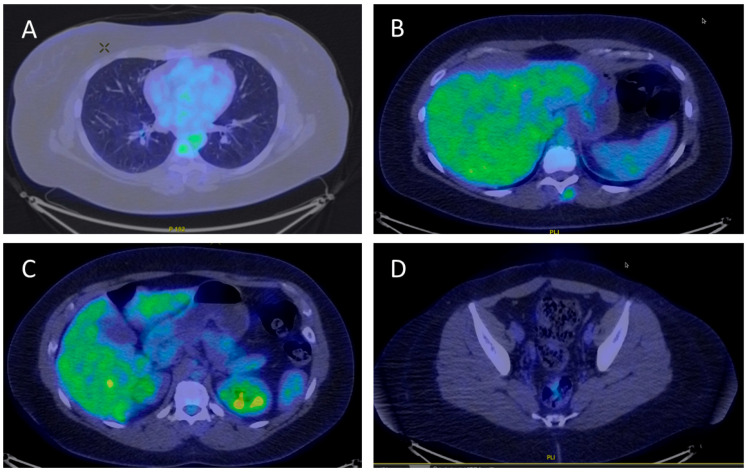
PET-CT images from 09/2024. No bright spots detected at the former metastatic sites. (**A**) Total regression of the pulmonary nodes. (**B**,**C**) No hepatic or splenic metastasis detected. (**D**) No pelvic metastasis detected. GE Signa^TM^ Explorer 1.5T (GE Health Care, 500 West Monroe Street, Chicago, IL 60661, USA) was used.

**Figure 4:**
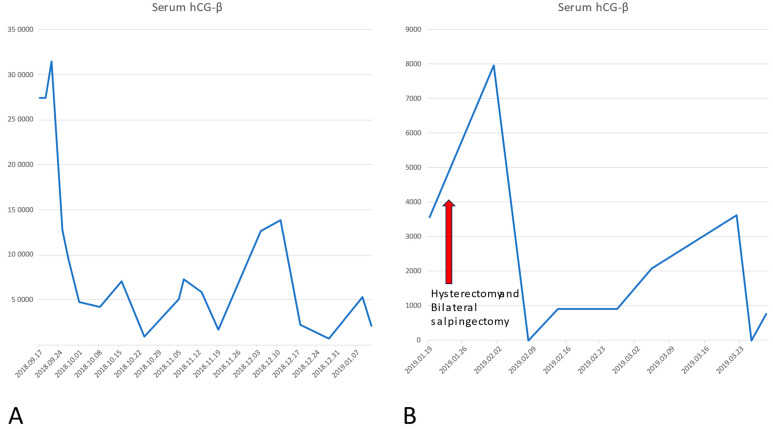
We can observe the changes in serum hCG-B during the clinical course of the disease. The changes in the serum hCG cover a wide range. For better presentation, we changed the scale of the “Y” axis at 19 January 2019. (**A**) Concentration of serum hCG from the diagnosis until January 2019. (**B**) Changes in serum hCG after the hysterectomy and bilateral salpingectomy. Despite the multiagent chemotherapy induced, a plateau of serum hCG-B persisted.

**Figure 5:**
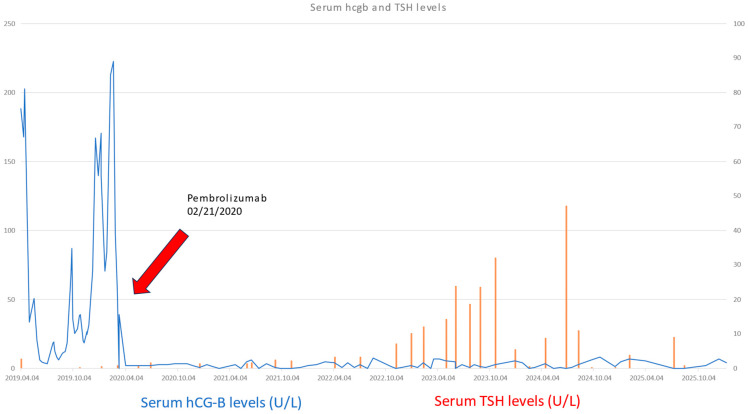
We can observe the significant remission and following suppression of serum hCG-β after the introduction of pembrolizumab. After 3 years of treatment, the patient developed hypothyroidism, which was successfully treated with hormone substitution.

In the past five years, the patient showed total remission based on serum hCG-β levels in the normal range and CAT scans and PET-CT showing remission of all metastasis (see Figure 3, Figure 4 and Figure 5). Though there were minimally elevated hCG-β levels detected around 6–7 U/L, this resulted to be false positive measurements due to serum hCG-β and luteinizing hormone (LH) cross-reaction [8]. Endocrine toxicity is a very common adverse effect of immune checkpoint inhibitors. Autoimmune thyroiditis is often observed during treatment with pembrolizumab [9]. The patient presented elevated levels of thyroid stimulating hormone (TSH) after two and a half years of treatment with PD-1 inhibitor pembrolizumab. After consultation with an endocrinologist, the condition was successfully treated with hormone replacement.

## 3. Discussion and Conclusions

Gestational trophoblastic neoplasia includes a group of relatively rare conditions, making the early diagnostics difficult. It is pivotal to organize bigger regional and national centrums that can provide diagnostic and therapeutic support. The low case number presented in smaller OBGYN units makes the early detection complicated, often causing the delay in appropriate treatment, leading to increased costs in both financial and health resource expenditures. Based on the WHO risk score system, surgical therapy and adjuvant chemotherapy can be used. In the majority of the cases, surgery combined with multiagent chemotherapy can be effective in treating even high-risk disease patients. Successful salvage therapy with *pembrolizumab* is challenging because of the limited experience due to the relatively low number of suitable candidates. In our paper, we reported the first successful pembrolizumab therapy in multichemotherapeutic-agent-resistant choriocarcinoma. The serum hCG-β levels significantly dropped after the initiation of pembrolizumab and remained so as of January 2026 (time period covered by our paper). The regression of the disease was also detected via imaging diagnostics. As frequently occurs, according to the literature, hypothyroidism was developed due to the PD-1 blocking therapy, which was successfully corrected with thyroid hormone substitution. Effective treatment of chemoresistant tumors may require inter-institutional co-operation. However, this case offers an encouraging example for future candidates for immunotherapy.

## Data Availability

Data can be found in the Hungarian National medical inform data base (EESZT) and can be provided if requested.

## Abbreviations

The following abbreviations are used in this manuscript:

GTN	Gestational trophoblastic neoplasia
GTD	Gestational trophoblastic disease
PD-L	Programmed death ligand
EMA-CO	Etoposide, methotrexate, actinomycin-d, cyclophosphamide, vincristine
EP- EMA	Etoposide, cisplatin, methotrexate, actinomycin-D
TP/TA	Paclitaxel, cisplatin, etoposide
